# Platelet aggregation and its modulation using antithrombotic agents

**DOI:** 10.6026/973206300210357

**Published:** 2025-03-31

**Authors:** Nandini Srivastava, Kaushik Ghanshyambhai Khatrani, Bansari Yagnik Tank, Yagnik Prafulchandra Tank, Urvashi Bachwani, Kevin Ashokbhai Purohit

**Affiliations:** 1 Department of Physiology, Maharshi Vashishtha Autonomous State Medical College, Basti, Uttar Pradesh, India; 2 Department of Physiology, GMERS Medical College, Himmatnagar, Gujarat, India; 3 Department of Microbiology, Dr. N. D. Desai Faculty of Medical Science and Research, Dharmsinh Desai University, Nadiad, Gujarat, India; 4 Department of Pathology, Dr. N. D. Desai Faculty of Medical Science and Research, Dharmsinh Desai University, Nadiad, Gujarat, India; 5 Department of Physiology, GMERS Medical College, Vadnagar, Gujarat, India; 6 Department of Physiology, GMERS Medical College, Vadnagar, Gujarat, India

**Keywords:** Platelet aggregation, antithrombotic agents, ticagrelor derivative, rivaroxaban analog, apixaban variant, thrombosis, cardiovascular diseases, light transmission aggregometry

## Abstract

Aggregation of platelets using three antithrombotic agents such as Ticagrelor Derivative (TD-101), Rivaroxaban Analog (RA-202) and Apixaban Variant (AV-303) were assessed. All the three agents showed dose-dependent inhibitory effects in platelet aggregation where AV-303 exhibited the strongest inhibitory activity according to light transmission aggregometry. The statistical analysis showed distinctions (p < 0.05) between groups receiving treatment and controls. This opens possibilities for medical applications in thrombotic disorder treatment. It should be noted that additional animal tests and clinical studies are needed to validate effectiveness and safety of these agents.

## Background:

Cardiovascular diseases (CVDs) represent among the main global causes of premature death and illness whereas thrombotic events significantly contribute to disease development [1]. Activated platelets following adherence to the vascular endothelium form platelet-rich thrombi through aggregation that causes arterial blockage and generates ischemic complications [2]. Inhibiting platelet aggregation stands as a fundamental therapeutic method to control and stop thrombotic disorders since it assists in treating myocardial infarction and stroke besides deep vein thrombosis [3]. Modern antithrombotic medications consist of antiplatelet drugs including aspirin and P2Y12 receptor inhibitors (clopidogrel, prasugrel and ticagrelor) besides anticoagulants that include heparin, warfarin together with direct oral anticoagulants (DOACs) such as rivaroxaban and apixaban [4, 5]. Despite their effectiveness antithrombotic drugs present restrictions that encompass inconsistent patient reactions together with enhanced bleeding susceptibility and drug-resistant problems [6]. The search for new antithrombotic drugs has become continuous because better safety and enhanced efficacy remain essential goals for drug development. The pharmaceutical sector now prioritizes developing new antithrombotic agent derivatives which offer improved absorption and action traits [7]. Apixaban is superior to warfarin in preventing stroke or systemic embolism, with less bleeding and lower mortality in patients with atrial fibrillation [8]. Recent research demonstrates that rivaroxaban along with apixaban acts as factor Xa inhibitor which effectively decreases thrombotic events yet these drugs generate continued bleeding concerns at different dosage levels [9, 10]. There is significant variability in individual responses to clopidogrel therapy for coronary stenting, with many patients showing inadequate platelet inhibition and some remaining resistant over time. High baseline platelet activity is associated with reduced effectiveness of the medication, highlighting the need for alternative treatment approaches and further exploration of the connection between poor drug response and adverse outcomes [11]. Therefore, it is of interest to develop improved and safer agents to prevent thrombotic disorders clinically.

## Materials and Methods:

A laboratory-based experimental research evaluated the impact of newly developed antithrombotic drugs on platelet aggregation. Healthy non-smokers participated in this study by giving venous blood donations since they remained free from antiplatelet and anticoagulant medications for two weeks prior to testing. A 21-gauge needle collected the venous blood which was placed in sodium citrate (3.2%) anti-coagulated tubes at a blood-to-anticoagulant ratio of 9:1. Shelf-ready blood samples distributed into PRP and PPP using 150 x g centrifugation at room temperature during a 10-minute spinning cycle obtained the PRP fraction. PPP was isolated by subjecting residual blood to 2,500 x g for 15 minutes because this plasma would serve as the standard reference during aggregometry tests. The measurement of platelet aggregation involved performing light transmission aggregometry (LTA) using a dual-channel aggregometer device. The researchers adjusted Platelet Rich Plasma to a platelet concentration of 250,000/µL through the addition of Poor Platelet Plasma. Light transmission measurements occurred during 10 minutes while ADP concentration reached 10 µM to induce platelet aggregation in the solution. The research examined three newly developed antithrombotic agents TD-101 Ticagrelor Derivative and both RA-202 Rivaroxaban Analog and AV-303 Apixaban Variant using concentrations of 1 µM, 5 µM as well as 10 µM. Each PRP solution received testing treatment with respective drug compounds during a five-minute period before ADP stimulation. The study included:

[1] The experimental group consisted of PRP exposed to ADP along with the absence of test compound.

[2] Treatment groups: PRP with ADP and one of the three compounds at different concentrations.

The percentage of light transmission served as a measurement metric throughout the experiment using PPP for full transmission but PRP showed no transmission. Scientists obtained aggregation measurements at their highest values for all tested samples. Data analysis occurred through one-way analysis of variance (ANOVA) and post hoc Tukey's test established which groups demonstrated statistically significant differences. The study determined statistical significance at less than 0.05 p-values. The results appeared as the mean value ± standard deviation (SD) measurement. The researchers conducted their experiments three times to confirm reliability while applying SPSS software version 26.0 for statistical analysis.

## Results:

All three newly synthesized compounds including Ticagrelor Derivative (TD-101), Rivaroxaban Analog (RA-202) and Apixaban Variant (AV-303) displayed dose-dependent results in the *in vitro* platelet aggregation studies. Tests on the control group established ADP as an effective agonist since it produced platelet aggregation results of 92.5 ± 3.2%. Evaluation results demonstrated the maximum inhibitory potency belonged to AV-303 followed by TD-101 and RA-202 among the tested compounds. At a concentration of 1 µM, AV-303, TD-101 and RA-202 reduced platelet aggregation by 30.2 ± 2.5%, 25.5 ± 3.1% and 18.3 ± 2.8%, respectively. The inhibition capability of AV-303 reached 55.1 ± 3.4% at 5 µM concentration while TD-101 exhibited 48.6 ± 2.9% and RA-202 demonstrated 42.2 ± 3.0% inhibition. When exposed to 10 µM concentration of AV-303, the inhibitory effect reached 80.3 ± 2.7% with TD-101 inhibiting at 70.5 ± 3.6% and RA-202 at 65.8 ± 2.9%. A statistical analysis revealed significant differences between all treated groups along with their control group (p < 0.05). Table 1 summarizes the inhibitory effects of the test compounds at different concentrations. The tests showed that among all tested compounds AV-303 exhibited the greatest efficacy for antithrombotic effects because it produced substantial platelet inhibition at its maximum concentration of 10 µM (Table 1). All tested concentrations of AV-303 produced meaningful differences (p < 0.05) when compared to other compounds according to statistical analysis. The lab results show that platelet aggregation responded inversely to compound concentration with AV-303 proving strongest first then TD-101 with RA-202 showing the next most potent effects. Research data demonstrates the clinical worth of these substances for treating thrombotic disorders.

## Discussion:

This research on anti-coagulation agents *in vitro* showed that TD-101 Ticagrelor Derivative and RA-202 Rivaroxaban Analog and AV-303 Apixaban Variant inhibited platelet aggregation based on injected dose levels. The laboratory investigation revealed that AV-303 displayed optimal inhibitory effects which imply its capacity to lead thrombotic disorder management more effectively. Study findings agree with established knowledge about platelet aggregation studies in heart diseases alongside the necessity to create better antithrombotic agents with enhanced safety profiles [1, 2]. The prevention and treatment of thrombotic conditions use three conventional antithrombotic drugs which include aspirin and P2Y12 inhibitors and direct oral anticoagulants (DOACs) [3]. Clinical use of reversible P2Y12 inhibitor ticagrelor remains limited because patients experience dyspnea and bleeding complications alongside its superior platelet inhibitory effect compared to clopidogrel [4, 5]. How rivaroxaban and apixaban function as factor Xa inhibitors to prevent thromboembolic events whereas their bleeding risks are directly proportionate to dose administration [6, 7]. The experimental data from this research demonstrate TD-101 combined with RA-202 as well as AV-303 could introduce a less harmful therapeutic method for management. The most effective inhibitor AV-303 achieved an 80.3% reduction in platelet aggregation when used at 10 µM and outperformed the available clinical drugs ticagrelor and prasugrel [8]. This research suggests that the new compound has potential use in clinical settings for delivering strong platelet inhibition without causing insufficient bleeding risks. The observed better inhibition potential of AV-303 depends on receptor binding efficiency and prolonged half-life duration that requires additional pharmacokinetic tests [9].

The inhibitory effects of the three compounds on platelet aggregation increased with their dosage levels thus making them suitable antithrombotic candidates. The ability to customize dosing through patient-specific risk variables enables medical professionals to minimize adverse bleeding effects known to occur with standard drug doses [10]. Importantly, patients with higher pre-treatment platelet reactivity continued to show higher levels of reactivity even after treatment, indicating they were less effectively protected by clopidogrel. These findings highlight the need for alternative pharmacological strategies and further investigation into the relationship between inadequate platelet inhibition and adverse ischemic events in these patients [11]. Previous research reinforces how doctors should use individual assessment of patients to choose specific antithrombotic drugs because this method helps manage the benefits of preventing clots against bleeding risks [12, 13]. Research outcomes demonstrate the medical usefulness of these new compounds that might be useful for patient-specific anticoagulation protocols.

Additional research needs to explore what factors lead to improved performance of these newly developed agents. Receptor binding together with bioavailability improvement due to structural arrangements in TD-101, RA-202 and AV-303 enhances their potency over original compounds [14]. The projected research should dedicate attention to pharmacodynamic and pharmacokinetic investigations together with *in vivo* tests on animal models to enable advancement toward clinical trials. These new compounds require complete evaluation of their drug interactions with all medications because cardiovascular disease polypharmacy treatments commonly utilize multiple medicines [15]. This research delivers important findings regarding the platelet aggregation changes from new antithrombotic drugs but still encounters specific research limitations. This research utilized an *in vitro* approach without properly mimicking the full human circulatory conditions. Inter-individual differences in platelet functioning have not been factored into the analysis even though such variations could affect the clinical performance of these compounds. Further ex vivo and *in vivo* testing must confirm these results for the agents to advance toward clinical implementation. Advances in antithrombotic therapy are moving closer to achieving safer treatments that effectively prevent clots without increasing bleeding risk [16].

## Conclusion:

The new antithrombotic agents TD-101, RA-202 and AV-303 exhibited strong platelet aggregation-blocking properties in a manner that grew more pronounced with each increasing dose until AV-303 demonstrated the greatest inhibitory effect. These agents hold promise to act as new therapeutic choices for treating thrombotic diseases among patients who need protection against excessive bleeding associated with standard treatment approaches. Pharmacokinetic analysis of these agents alongside *in vivo* experiments and clinical trial assessments will be required to define their efficacy standards in actual medical care.

## Figures and Tables

**Table 1:** Effect of Antithrombotic Agents on Platelet Aggregation (%)

**Group**	**1 µM (%)**	**5 µM (%)**	**10 µM (%)**
Control (ADP only)	92.5 ± 3.2	92.5 ± 3.2	92.5 ± 3.2
TD-101	25.5 ± 3.1	48.6 ± 2.9	70.5 ± 3.6
RA-202	18.3 ± 2.8	42.2 ± 3.0	65.8 ± 2.9
AV-303	30.2 ± 2.5	55.1 ± 3.4	80.3 ± 2.7
(Values are expressed as mean ± SD. All test compounds
showed significant inhibition compared to the control group, p < 0.05.)
